# IL‐6 counteracts the inhibitory effect of IL‐4 on osteogenic differentiation of human adipose stem cells

**DOI:** 10.1002/jcp.28652

**Published:** 2019-04-23

**Authors:** Angela P. Bastidas‐Coral, Jolanda M. A. Hogervorst, Tymour Forouzanfar, Cornelis J. Kleverlaan, Pieter Koolwijk, Jenneke Klein‐Nulend, Astrid D. Bakker

**Affiliations:** ^1^ Department of Oral Cell Biology Academic Centre for Dentistry Amsterdam (ACTA), University of Amsterdam and Vrije Universiteit Amsterdam, Amsterdam Movement Sciences Amsterdam The Netherlands; ^2^ Department of Oral and Maxillofacial Surgery Amsterdam University Medical Centers (Amsterdam UMC)/ACTA, location VUmc, Amsterdam Movement Sciences Amsterdam The Netherlands; ^3^ Department of Dental Materials Science Academic Centre for Dentistry Amsterdam (ACTA), University of Amsterdam and Vrije Universiteit Amsterdam, Amsterdam Movement Sciences Amsterdam The Netherlands; ^4^ Department of Physiology Amsterdam Cardiovascular Sciences, Amsterdam University Medical Centers (Amsterdam UMC) Amsterdam The Netherlands

**Keywords:** hASCs, interleukin‐4, interleukin‐6, osteogenic differentiation, oxygen tension

## Abstract

Fracture repair is characterized by cytokine production and hypoxia. To better predict cytokine modulation of mesenchymal stem cell (MSC)‐aided bone healing, we investigated whether interleukin 4 (IL‐4), IL‐6, and their combination, affect osteogenic differentiation, vascular endothelial growth factor (VEGF) production, and/or mammalian target of rapamycin complex 1 (mTORC1) activation by MSCs under normoxia or hypoxia. Human adipose stem cells (hASCs) were cultured with IL‐4, IL‐6, or their combination for 3 days under normoxia (20% O _2_) or hypoxia (1% O _2_), followed by 11 days without cytokines under normoxia or hypoxia. Hypoxia did not alter IL‐4 or IL‐6‐modulated gene or protein expression by hASCs. IL‐4 alone decreased runt‐related transcription factor 2 (RUNX2) and collagen type 1 (COL1) gene expression, alkaline phosphatase (ALP) activity, and VEGF protein production by hASCs under normoxia and hypoxia, and decreased mineralization of hASCs under hypoxia. In contrast, IL‐6 increased mineralization of hASCs under normoxia, and enhanced RUNX2 gene expression under normoxia and hypoxia. Neither IL‐4 nor IL‐6 affected phosphorylation of the mTORC1 effector protein P70S6K. IL‐4 combined with IL‐6 diminished the inhibitory effect of IL‐4 on ALP activity, bone nodule formation, and VEGF production, and decreased RUNX2 and COL1 expression, similar to IL‐4 alone, under normoxia and hypoxia. In conclusion, IL‐4 alone, but not in combination with IL‐6, inhibits osteogenic differentiation and angiogenic stimulation potential of hASCs under normoxia and hypoxia, likely through pathways other than mTORC1. These results indicate that cytokines may differentially affect bone healing and regeneration when applied in isolation or in combination.

## 1 INTRODUCTION

Small bone injuries can heal spontaneously without intervention of an orthopedic surgeon. However, trauma, infection, congenital disorders, or tumor resection, sometimes lead to nonhealing large bone defects. Despite the development of new orthopedic devices and advanced techniques, these defects are difficult to treat and still represent major challenges in orthopedic surgery (Guerado & Caso, 2017; Verrier et al., 2016). Bone tissue engineering techniques using a combination of progenitor cells seeded on osteoconductive scaffolds with osteoinductive growth factors, are the current strategy of choice for the treatment of large bone defects (Ho‐Shui‐Ling et al., 2018). Mesenchymal stem cells (MSCs) are frequently used for tissue engineering purposes, in particular adipose‐derived stem cells (ASCs) represent a promising source due to their abundance, accessibility, and osteogenic differentiation potential (Farre‐Guasch et al., 2018; Prins, Schulten, Ten Bruggenkate, Klein‐Nulend, & Helder, 2016). However, little is known about the conditions favouring the osteogenic potential of MSCs. Mimicking some aspects of the physiological conditions of bone healing will lead to the development of therapies to aid bone repair in large defects that are currently difficult to treat.

Oxygen tension is low in or near fracture sites (Chung, Won, & Sung, 2009; Lu et al., 2013). Hypoxia has been reported to increase ASC proliferation, stemness marker expression, and chondrogenic differentiation, but to reduce adipogenic and osteogenic differentiation potential (Choi et al., 2014). It has also been reported that hypoxia promotes chondrogenic, osteogenic, and adipogenic differentiation in vitro, and that hypoxic cells show an increased bone repairing capacity in vivo (Tsai et al., 2011).

Bone injury initially causes blood vessel disruption, resulting in a hypoxic environment, followed by hematoma formation and an inflammatory response. During this inflammatory response, different proinflammatory and anti‐inflammatory cytokines are released to the injury site (Loi et al., 2016; Mountziaris & Mikos, 2008). For instance, the proinflammatory cytokine interleukin‐6 (IL‐6) is known to be released in the first 72 hr after bone fracture and to rapidly decline thereafter (Ai‐Aql, Alagl, Graves, Gerstenfeld, & Einhorn, 2008; Einhorn, Majeska, Rush, Levine, & Horowitz, 1995). IL‐6 is secreted by multiple cell types, such as osteoblasts, and stimulates osteoclast formation and bone resorption, thereby playing a role in bone homeostasis (Kudo et al., 2003; Majumdar, Thiede, Haynesworth, Bruder, & Gerson, 2000). However, the exact role of IL‐6 during fracture healing is still unknown, even though IL‐6 knockout mouse studies indicate that lack of IL‐6 delays bone healing (Yang et al., 2007).

The T helper type 2 cytokine IL‐4 is also present during fracture healing (Toben et al., 2011), and is considered anti‐inflammatory as it inhibits the production of IL‐1, tumor necrosis factor α (TNF‐α), and prostaglandin E_2_ by monocytes (Baumann & Gauldie, 1994). IL‐4 also inhibits bone resorption (Watanabe et al., 1990), and is a chemoattractant for osteoblasts (Lind, Deleuran, Yssel, Fink‐Eriksen, & Thestrup‐Pedersen, 1995). However, the exact role of IL‐4 during fracture healing is still unclear. We have shown earlier that treatment with IL‐4 or IL‐6 during 72 hr exerts opposite effects on osteogenic differentiation of MSCs, that is, IL‐6 stimulates, but IL‐4 inhibits osteogenic differentiation (Bastidas‐Coral et al., 2016). In addition, little is known about the stimulatory or inhibitory effects of the combination of proinflammatory and anti‐inflammatory cytokines in a hypoxic environment, as occurs during bone healing, on the osteogenic differentiation potential of MSCs. This represents a significant hiatus in our knowledge, considering that (a) cytokines as well as hypoxia are hallmarks of the early stages of fracture healing, (b) both cytokines and hypoxia will likely be present in any implanted tissue‐engineered construct in vivo, and (c) cytokines and hypoxia possibly interact at the level of signal transduction. A previous study from our group has also demonstrated the importance of studying the effect of different cytokines in combination, showing that the combination of cytokines present in the circulation of patients with active rheumatoid arthritis might contribute to generalized bone loss by directly inhibiting osteoblast proliferation and differentiation (Pathak et al., 2014).

Under hypoxia mammalian target of rapamycin (mTOR) is inactivated, which may occur as part of the cell program to maintain energy homeostasis (Knaup et al., 2009). IL‐6 is known to signal via GP130/IL‐6R, activating the JAK/STAT pathway, which activates the mTOR pathway via insulin‐like growth factor 1. The mammalian target of rapamycin complex 1 (mTORC1) signaling pathway is required for osteoblast proliferation and differentiation (Singha et al., 2008), and it plays an important role in the regulation of bone metabolism and skeletal development by regulating messenger RNA (mRNA) translation during preosteoblast differentiation (Bakker & Jaspers, 2015; Fitter et al., 2017). The activation of IL‐4 signaling in bone marrow MSCs (BMMSCs) favors adipogenic differentiation and prevents osteoblast differentiation in an mTORC1‐dependent manner in a mutant “tight skin” mouse model of systemic sclerosis (Chen et al., 2015). Thus, hypoxia, IL‐4, and IL‐6 pathways may interact at the level of mTORC1. Interestingly, both the osteogenic effect of IL‐6 on human BMSCs and the antiosteogenic effect of IL‐4 on BMMSCs have been ascribed to mTORC1 activation (Chen et al., 2015; Deshpande et al., 2013).

The aim of this study was to determine whether IL‐4, IL‐6, or the combination of both cytokines affects osteogenic differentiation and the angiogenic stimulation potential of MSCs under normoxia and hypoxia. We hypothesized that IL‐4 decreases, while IL‐6 enhances osteogenic differentiation and vascular endothelial growth factor (VEGF) production, and that the combination of both cytokines will not enhance nor decrease osteogenic differentiation and VEGF production in MSCs, as the effect of IL‐4 and IL‐6 will counterbalance. The osteogenic and angiogenic effects exerted by IL‐4 and/or IL‐6 will be accompanied by mTORC1 activation in MSCs.

## 2 MATERIALS AND METHODS

### 2.1 Adipose tissue donors

Subcutaneous adipose tissue samples were harvested from abdominal wall resections of five healthy female donors (age range, 33–54 years; mean, 47 years), who underwent elective plastic surgery at the Tergooi Hospital Hilversum and a clinic in Bilthoven, The Netherlands. The Ethical Review Board of the VU Medical Center, Amsterdam, The Netherlands, approved the protocol and informed consent was obtained from all patients.

### 2.2 Isolation and culture of human adipose stem cells (hASCs)

Isolation, characterization, and osteogenic differentiation capacity of hASCs has been previously reported by our group (Varma et al., 2007). For the isolation of hASCs, adipose tissue was cut into small pieces and enzymatically digested with 0.1% collagenase A (Roche Diagnostics GmbH, Mannheim, Germany) in phosphate‐buffered saline (PBS) containing 1% bovine serum albumin (Roche Diagnostics GmbH) under continuous stirring for 45 min at 37°C. Next, Ficoll® density‐centrifugation step (Lymphoprep™; 1,000*g*, 20 min, *ρ* = 1.077 g/ml Ficoll®, osmolarity 280 ± 15 mOsm; Axis‐Shield, Oslo, Norway) was performed, and the resulting stromal vascular fraction pellet containing ASCs was resuspended in Dulbecco's modified Eagle's medium (DMEM; Life Technologies Europe BV, Bleiswijk, The Netherlands). Finally, cells were counted and stored in liquid nitrogen. Cryopreserved stromal vascular fraction‐containing ASC suspensions from the different donors were pooled and cultured in α‐minimum essential medium (α‐MEM; Gibco, Life Technologies, Waltham, MA) with 1% penicillin, streptomycin, and fungizone (PSF; Sigma‐Aldrich , St Louis, MO), 10 IU/ml heparin (Leo Pharma A/S, Ballerup, Denmark) and 5% human platelet lysate (PL) at 37°C in a humidified atmosphere with 5% CO_2_. The medium was refreshed two times a week. When near confluent (90%), cells were harvested by adding 0.25% trypsin (Gibco, Invitrogen), and 0.1% ethylenediaminetetraacetic acid (Merck, Darmstadt, Germany) in PBS at 37°C, replated, cultured until passage 2 (P2), and stored in liquid nitrogen until further use. For experiments, cryopreserved pooled hASCs were thawed and seeded at 0.5 × 10^6^ cells in T‐175 cm^2^ culture flasks (Greiner Bio‐One, Kremsmuenster, Austria) in αMEM containing 1% PSF, 10 IU/ml heparin, and 2% human PL in a humidified atmosphere with 5% CO_2_ at 37°C. In all experiments, hASC at P3 were used. Medium was changed every 3 days.

### 2.3 Platelet lysate

Pooled platelet products from five donors were obtained from the Bloodbank Sanquin (Sanquin, Amsterdam, The Netherlands) and contained approximately 1 × 10^9^ platelets/ml (Prins et al., 2009). PL was obtained by lysing the platelets through temperature shock at −80°C. Before use, PL was thawed and centrifuged at 600*g* for 10 min to eliminate remaining platelet fragments. The supernatant was added at 2% (v/v) to the medium.

### 2.4 Stimulation of hASCs with IL‐4 and/or IL‐6

HASCs (10 × 10^3^ cells/cm^2^) were seeded into 24‐wells plates and cultured in αMEM containing 1% PSF, 10 IU/ml heparin, and 2% human PL in a humidified atmosphere containing 20% O_2_ (normoxia [standard condition]) or 1% O_2_ (hypoxia), and allowed to attach for 24 hr at 37°C. The next day, the medium was replaced with osteogenic medium, consisting of αMEM with 1% PSF, 10 IU/ml heparin, 2% human PL, 50 µM ascorbic acid‐2‐phosphate (vitamin C; Sigma‐Aldrich), 5 mM β‐glycerophosphate (Sigma‐Aldrich) and 10 nM 1,25‐(OH)_2_vitamin D_3_ (Sigma‐Aldrich). Recombinant human IL‐4 (R&D Systems, Minneapolis, MN), and/or recombinant human IL‐6 (R&D Systems) and recombinant human IL‐6Rα (R&D Systems) were added to the osteogenic medium. IL‐4 or IL‐6 were added in a final concentration of 1 and 10 ng/ml, respectively, and IL‐4 in combination with IL‐6 in a final concentration of 10 ng/ml. After addition of osteogenic medium supplemented with the cytokines, hASCs were incubated in 1% O_2_ or 20% O_2_ at 37°C during 3 days. Then, the medium was replaced by osteogenic medium without cytokines, and refreshed every 3 days during 11 days. hASCs were harvested after 2, 7, and 14 days of culture for analysis of proliferation, osteogenic differentiation, and VEGF expression as described below.

### 2.5 hASCs culture under hypoxia

HACSs were cultured under hypoxia inside a custom designed hypoxic workstation (Top Class Products and Services, Rotselaar, Belgium), where oxygen concentration was controlled via injection of N_2_ as described (Nauta, Duyndam, Weijers, van Hinsbergh, & Koolwijk, 2016). Oxygen concentration inside the incubator was continuously monitored with an internal zirconia sensor, as well as by periodically external calibration with O_2_ test tubes (Drager Safety, Zoetermeer, The Netherlands). To maintain the hypoxic condition of the hASCs, medium was preincubated for 3 hr under hypoxia before use. Hypoxia was defined as 1% O_2_, 5% CO_2_, and 94% N_2_.

### 2.6 DNA content

HASC cultured for 2 and 7 days with IL‐4, IL‐6, or both (IL‐4 + IL‐6), were washed with PBS, and lysis buffer was added. DNA content as a measure for cell number was determined using a Cyquant Cell Proliferation Assay Kit (Molecular Probes, Leiden, The Netherlands). Absorption was read at 485 nm excitation and 528 nm emission in a microplate reader (Synergy™ HT spectrophotometer; BioTek Instruments Inc, Highland Park, Winooski, VT).

### 2.7 RNA isolation and real‐time reverse transcription polymerase chain reaction (RT‐PCR)

RNA isolation from hASCs was performed using the RNeasy Mini Kit (74106; Qiagen, Venlo, The Netherlands) according to the manufacturer's instructions. RNA concentration and quality was measured using a Synergy HT spectrophotometer. RNA was reverse‐transcribed to complementary DNA (cDNA) using a RevertAid™ First Strand cDNA Synthesis Kit (Fermentas, St. Leon‐Rot, Germany) according to manufacturer's instructions. The cDNA was diluted to a final concentration of 2 ng/μl. Real‐time PCR was performed using the SYBR® Green I Mastermix (Roche Diagnostics, Mannheim, Germany) in a LightCycler® 480 (Roche Diagnostics, Basel, Switzerland). Every PCR reaction was prepared with 4 μl cDNA, 0.5 μl forward primer (1 μM), 0.5 μl reverse primer (1 μM), 5 μl LightCycler® 480 SYBR® Green I Mastermix (Roche Diagnostics, Mannheim, Germany) in a final volume of 10 μl. Based on BestKeeper (Pfaffl, Tichopad, Prgomet, & Neuvians, 2004), values were normalized to TATA‐box binding protein and β‐glucuronidase housekeeping genes. Real‐time PCR was used to assess gene expression of KI67, runt‐related transcription factor 2 (RUNX2), collagen type 1 (COL1), osteocalcin, and VEGF‐165. All primers used were from Life Technologies. The primer sequences are listed in Table 1. mRNA preparations of hASCs were used as a reference and internal control in each assay.

**Table 1:** List of primer sequences used for analysis of proliferation, osteogenic and angiogenic markers by hASCs by PCR

	Oligonucleotide sequences	
Genes (human)	Forward	Reverse
*TBP*	5′‐GGTCTGGGAAAATGGTGTGC‐3′	5′‐GCTGGAAAACCAACTTCTG‐3′
*GUSB*	5′‐CGCACAAGAGTGGTGCTGAG‐3′	5′‐GGAGGTGTCAGTCAGGTATT‐3′
*KI67*	5′‐CCCTCAGCAAGCCTGAGAA‐3′	5′‐AGAGGCGTATTAGGAGGCAAG‐3′
*RUNX2*	5′‐ATGCTTCATTCGCCTCAC‐3′	5′‐ACTGCTTGCAGCCTTAAAT‐3′
*COL1*	5′‐TCCGGCTCCTGCTCCTCTTA‐3′	5′‐GGCCAGTGTCTCCCTTG‐3′
*OC*	5′‐AGCCACCGAGACACCATGAGA‐3′	5′‐CTCCTGAAAGCCGATGTGGTC‐3′
*VEGF‐165*	5′‐ATCTTCAAGCCATCCTGTGTGC‐3′	5′‐CAAGGCCCACAGGGATTTTC‐3′

*Note*. COL1: collagen type 1; GUSB: β‐glucuronidase; KI67: proliferation marker; OC: osteocalcin; RUNX2: runt‐related transcription factor‐2; TBP: TATA‐box binding protein; VEGF‐165: vascular endothelial growth factor.

### 2.8 Alkaline phosphatase (ALP) activity

hASC cultured for 2, 4, and 7 days with IL‐4 and/or IL‐6 in 1% or 20% O_2_ were lysed with 250 µl milli‐Q water, and stored at −20°C until use. 4‐Nitrophenyl phosphate disodium salt (Merck) at pH 10.3 was used as a substrate for ALP, according to the method described by Lowry (Lowry, 1955). The absorbance was read at 405 nm with a Synergy HT spectrophotometer. ALP activity was expressed as µM/ng DNA.

### 2.9 Mineralization

Matrix mineralization was analyzed by alizarin red staining after incubation of hASCs with IL‐4 and/or IL‐6 in 1% or 20% O_2_ at Day 14 by using 2% Alizarin Red S (Sigma‐Aldrich) in water at pH 4.3, as described (Fukuyo et al., 2014). Briefly, hASCs were fixed with 4% formaldehyde for 15 min and rinsed with deionized water before adding 350 µl of the alizarin red solution per well. After incubation at room temperature for 30 min, the cells were washed with deionized water. Cells that have differentiated into osteoblasts deposit mineralized matrix, which is visible as bright red nodules. Quantification of the mineralized matrix was performed using ImageJ software 1.49 v (Wayne Rasband, National Institutes of Health, Bethesda, MD) as previously described (Shah et al., 2016).

### 2.10 VEGF quantification

VEGF protein concentration was measured in the supernatant of hASCs after incubation with IL‐4 and/or IL‐6 in 1% or 20% O_2,_ at Day 7, by using a Quantikine® Elisa kit (R&D Systems) according to the manufacturer's protocol. The absorbance was read at 450 nm with a microplate reader (Synergy HT spectrophotometer).

### 2.11 Western blot

hASCs were lysed in Ripa buffer to quantify total protein concentration with a bicinchoninic acid protein assay (Pierce, Rockford, IL). Homogenates were denatured by addition of sample buffer (final concentration: 50 mM Tris‐HCl (pH 6.8), 2% (w/v) sodium dodecyl sulfate, 10% (v/v) glycerol, and 2% β‐mercaptoethanol) followed by 5 min heating at 95°C. A total of 1.4 μg of protein in 15 μl sample buffer was separated on a 10% acrylamide gel (Bio‐Rad), and transferred during 2 hr in ice‐cold buffer onto a nitrocellulose membrane (GE Healthcare Life Sciences, Little Chalfont, UK). The separated proteins were transferred to a membrane and subjected to immunodetection. After overnight blocking in Amersham ECL prime blocking reagent (GE Healthcare, Amersfoort, The Netherlands), 1 hr incubations were carried out with rabbit primary antibody against either phospho P70S6K (pP70S6K at T389) or total P70S6K, and α‐tubulin in a dilution of 1/4,000 in blocking buffer. Membranes were washed in Tris‐buffered saline, 0.1% Tween 20 and incubated with rabbit‐specific polyclonal goat‐anti‐rabbit horseradish peroxidase‐conjugated secondary antibodies (Dako, Kopenhagen, Denmark) at a dilution of 1/4,000. The signal was detected with ECL Select Western Blotting Detection Reagent (GE Healthcare, Little Chalfont, UK) and recorded with an Image Quant LAS 5000 (GE Healthcare, Little Chalfont, Buckinghamshire, UK). Band intensities were quantified using ImageJ software 1.49 v (Wayne Rasband, National Institutes of Health).

### 2.12 Statistical analysis

Values are presented as mean ± standard deviation (*SD*). In total three independent experiments were performed in duplicate (*n* = 3) using IL‐4 or IL‐6 (1 and 10 ng/ml), and in triplicate (*n* = 3) using IL‐4 in combination with IL‐6 (10 ng/ml). Statistical significance was determined using analysis of variance, with application of Dunnett's multiple comparison test to compare IL‐4 or IL‐6 at 1 and 10 ng/ml with controls. Two‐tailed paired *t*‐test was used to compare control groups without cytokines under normoxia and hypoxia, and to compare IL‐4 in combination with IL‐6 with the control. A *p* < 0.05 was considered significant. Statistical analysis was performed using GraphPad Prism 5.4 (GraphPad Software, San Diego, CA).

## 3 RESULTS

### 3.1 hASC proliferation

**Figure 1:**
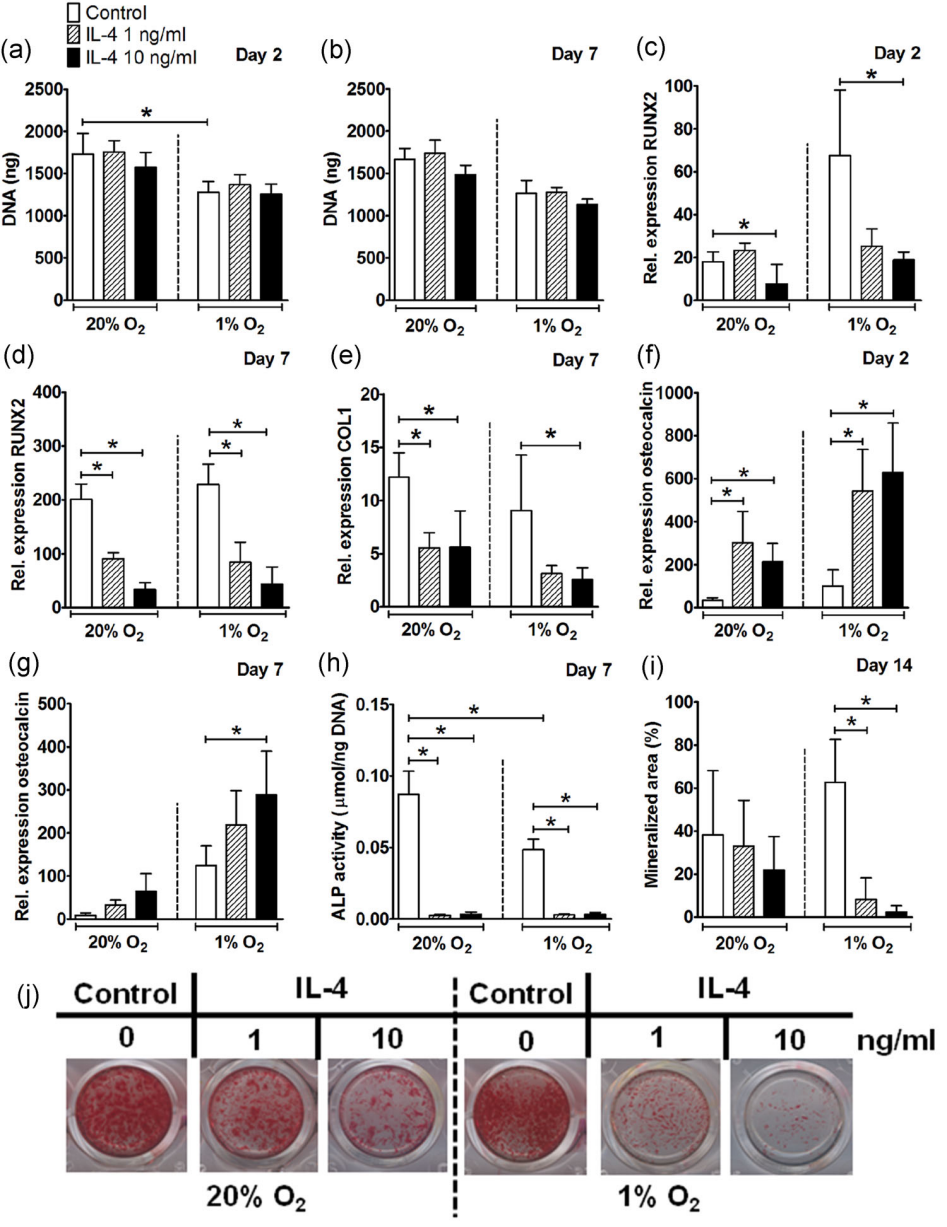
Effect of IL‐4 on proliferation and osteogenic differentiation of hASCs under normoxia or hypoxia. (a,b) Hypoxia decreased DNA content in hASCs cultured without IL‐4 at Day 2 but not at Day 7. IL‐4 did not affect DNA content in hASCs at Day 2 or 7. (c) IL‐4 at 10 ng/ml decreased RUNX2 expression under normoxia and hypoxia at Day 2. (d) IL‐4 at 1 and 10 ng/ml decreased RUNX2 expression under normoxia and hypoxia at Day 7. (e) IL‐4 decreased COL1 expression under normoxia (1 and 10 ng/ml), and under hypoxia (10 ng/ml) at Day 7. (f) IL‐4 at 1 and 10 ng/ml enhanced osteocalcin expression under normoxia and hypoxia at Day 2. (g) Under hypoxia, IL‐4 at 10 ng/ml enhanced osteocalcin expression at Day 7. (h) Hypoxia decreased ALP activity. IL‐4 (1 and 10 ng/ml) decreased ALP activity under normoxia and hypoxia at Day 7. (i) Quantification of mineralized matrix showed IL‐4 (1 and 10 ng/ml) to inhibit mineral deposition under hypoxia at Day 14. (j) IL‐4 at 1 and 10 ng/ml decreased matrix mineralization of hASCs under hypoxia, and to a lesser extend under normoxia at Day 14. Results are mean ± *SD* from *n* = 3 (duplicate wells of three independent experiments). *Significant effect of IL‐4 under normoxia or hypoxia, *p* < 0.05. ALP: alkaline phosphatase; COL1: collagen type 1; hASC: human adipose stem cell; IL: interleukin; RUNX2: runt‐related transcription factor 2 [Color figure can be viewed at wileyonlinelibrary.com]

**Figure 2:**
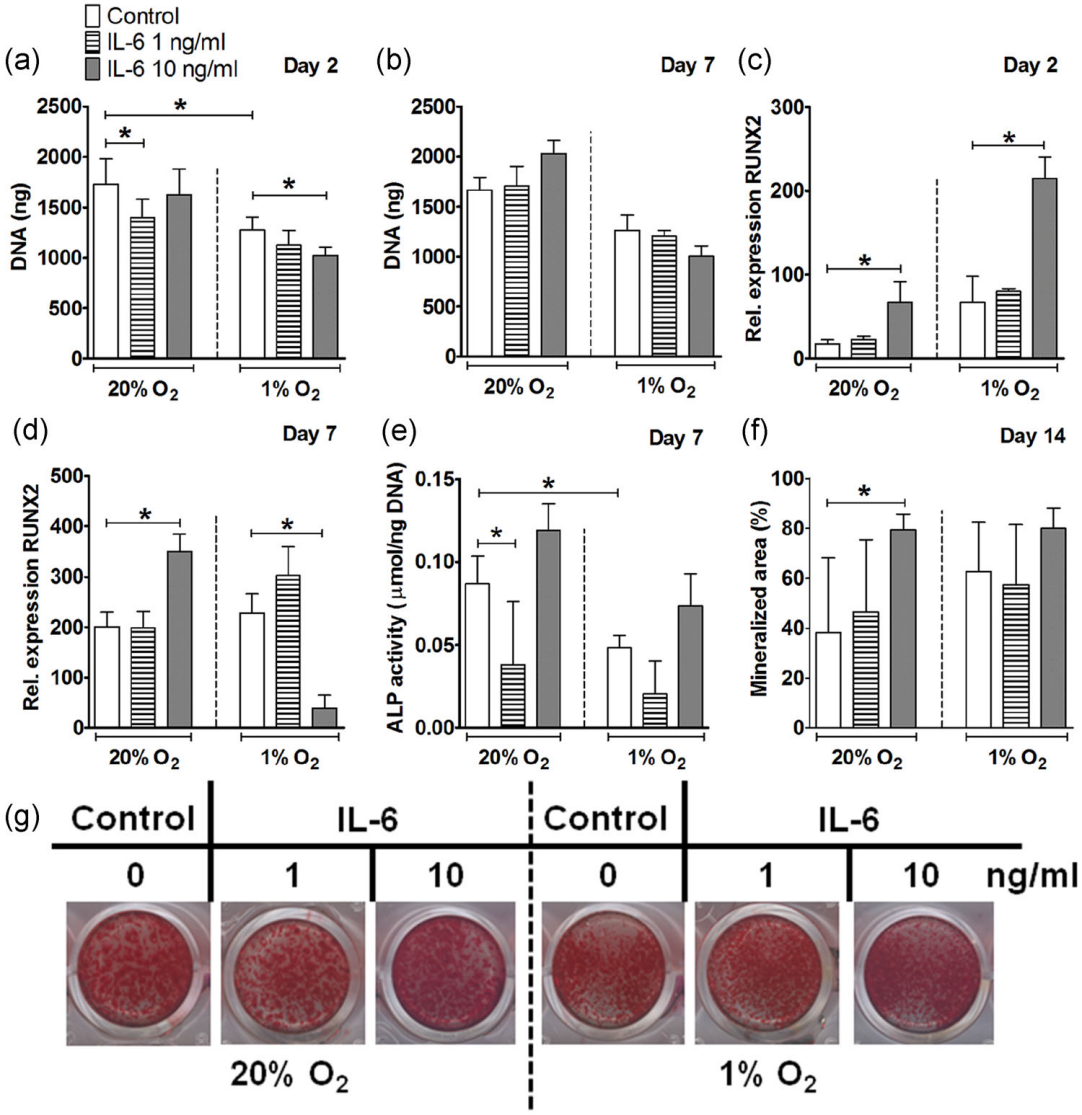
Effect of IL‐6 on proliferation and osteogenic differentiation of hASCs under normoxia or hypoxia. (a,b) Hypoxia decreased DNA content in hASCs cultured without IL‐6 at Day 2, but not at Day 7. Under normoxia, IL‐6 at 1 ng/ml reduced DNA content at Day 2. Under hypoxia, IL‐6 at 10 ng/ml reduced DNA content at Day 2 not at Day 7. (c) IL‐6 at 10 ng/ml increased RUNX2 expression under normoxia and hypoxia at Day 2. (d) At Day 7, IL‐6 at 10 ng/ml increased RUNX2 expression under normoxia and it decreased under hypoxia. (e) At Day 7, hypoxia decreased ALP activity. IL‐6 at 1 ng/ml decreased ALP activity under normoxia. (f) IL‐6 (10 ng/ml) enhanced mineralized area under normoxia at Day 14. (g) IL‐6 (1 and 10 ng/ml) induced mineralization of hASCs under normoxia and hypoxia at Day 14. Results are mean ± *SD* from *n* = 3 (duplicate wells of three independent experiments). *Significant effect of IL‐6 under normoxia or hypoxia, *p* < 0.05. ALP: alkaline phosphatase; COL1: collagen type 1; hASC: human adipose stem cell; IL: interleukin; RUNX2: runt‐related transcription factor 2 [Color figure can be viewed at wileyonlinelibrary.com]

**Figure 3:**
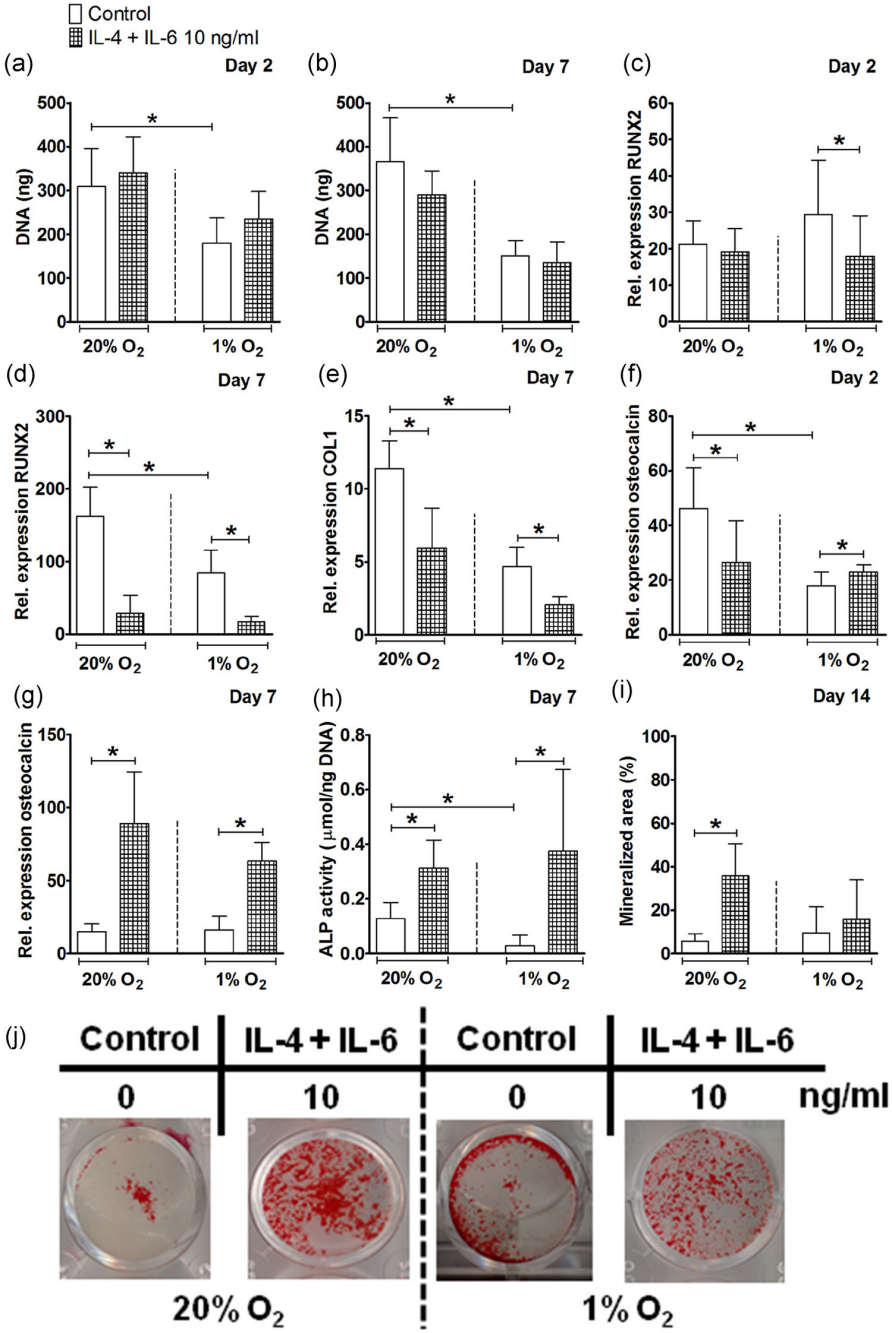
Effect of IL‐4 in combination with IL‐6 on proliferation and osteogenic differentiation of hASCs under normoxia or hypoxia. (a,b) Hypoxia decreased DNA content in hASCs cultured without the combination of IL‐4 with IL‐6 at Day 2 and 7. (c) The combination of cytokines decreased RUNX2 expression under hypoxia at Day 2. (d) At Day 7, hypoxia decreased RUNX2 expression. IL‐4 with IL‐6 decreased RUNX2 expression under normoxia and hypoxia. (e) At Day 7, hypoxia decreased COL1 expression. IL‐4 with IL‐6 decreased COL1 expression under normoxia and hypoxia. (f) At Day 2, hypoxia decreased osteocalcin expression. IL‐4 with IL‐6 decreased osteocalcin expression under normoxia and hypoxia. (g) IL‐4 with IL‐6 enhanced osteocalcin expression under normoxia and hypoxia at Day 7. (h) At Day 7, hypoxia decreased ALP activity. IL‐4 in combination with IL‐6 increased ALP activity under normoxia and hypoxia. (i) hASCs stimulated with IL‐4 in combination with IL‐6 showed increased mineralization under normoxia at Day 14. (j) IL‐4 with IL‐6 induced mineralization of hASCs under normoxia and hypoxia at Day 14. Results are mean ± *SD* from *n* = 3 (triplicate wells of three independent experiments). *Significant effect of IL‐4 in combination with IL‐6 under normoxia or hypoxia, *p* < 0.05. ALP: alkaline phosphatase; COL1: collagen type 1; hASC: human adipose stem cell; IL: interleukin; RUNX2: runt‐related transcription factor 2 [Color figure can be viewed at wileyonlinelibrary.com]

The effect of IL‐4, IL‐6, and IL‐4 in combination with IL‐6 on hASC number and proliferation under normoxia or hypoxia conditions was measured by quantification of DNA content. Stimulation with IL‐4 did not significantly affect DNA content under normoxia or hypoxia culture conditions at Day 2 or Day 7 (Figure 1a,b), while IL‐6 reduced DNA content by 1.2–1.3‐fold (1 ng/ml, normoxia;10 ng/ml, hypoxia, respectively) compared with controls at Day 2 (Figure 2a). Stimulation with the combination of IL‐4 and IL‐6 did not significantly affect DNA content under normoxia or hypoxia culture conditions at Day 2 or 7 (Figure 3a,b). Taken together, IL‐6 inhibited hASC DNA content, while IL‐4 and the combination of IL‐4 with IL‐6 did not. Next, we analyzed the effect of hypoxia, IL‐4, IL‐6, and IL‐4 combined with IL‐6 on markers of osteogenic differentiation and VEGF production in hASCs.

### 3.2 IL‐4 decreased osteogenic differentiation of hASCs under normoxia and hypoxia

Under normoxia, IL‐4 at 10 ng/ml decreased RUNX2 expression by 2.2–6.0‐fold at Day 2 and 7, and at 1 ng/ml by 2.2‐fold at Day 7 only. Under hypoxia, IL‐4 at 10 ng/ml decreased RUNX2 expression by 3.5–5.0‐fold at Day 2 and 7, and at 1 ng/ml by threefold at Day 7 (Figure 1c,d). COL1 gene expression in hASCs was also reduced by IL‐4 by 2.4‐fold under normoxia, and by 3.6‐fold under hypoxia at Day 7 (Figure 1e). IL‐4 did not affect COL1 gene expression at any dose or oxygen tension tested at Day 2 (data not shown). When osteocalcin gene expression by hASCs was analyzed, the opposite was found, that is, IL‐4 enhanced osteocalcin expression by 9.2‐fold under normoxia at Day 2, and to a lesser extend at Day 7, and by 2.3‐6.2**‐**fold under hypoxia at Day 2 and 7 (Figure 1f,g). IL‐4 almost abolished ALP activity in hASCs, as it reduced ALP activity by 24.4–38.4**‐**fold under normoxia, and by 16‐fold under hypoxia at Day 7 (Figure 1h). This inhibitory effect of IL‐4 on ALP activity was also reflected by decreased matrix mineralization by 7.6–25.7**‐**fold under hypoxia at Day 14, suggesting inhibition of osteogenic differentiation of hASCs under hypoxia compared with normoxia (Figure 1i,j). Taken together, IL‐4 strongly inhibited the expression of the osteogenic differentiation markers RUNX2 and COL1, as well as ALP activity and matrix mineralization of hASC.

### 3.3 IL‐6 enhanced bone nodule formation by hASCs under normoxia

IL‐6 at 10 ng/ml increased RUNX2 expression by 1.7–3.7‐fold under normoxia at Day 2 and 7, and by 3.2‐fold under hypoxia at Day 2, while IL‐6 decreased RUNX2 expression (5.8‐fold) under hypoxia at Day 7 (Figure 2c,d). Addition of IL‐6 at 1 ng/ml reduced ALP activity by 2.2‐fold under normoxic culture conditions at Day 7 (Figure 2e). In contrast, IL‐6 (10 ng/ml) significantly enhanced mineralized area (2.1‐fold) under normoxia at Day 14 (Figure 2f,g). IL‐6 did not affect COL1 or osteocalcin gene expression at any dose, time point, or oxygen tension tested (data not shown).

### 3.4 IL‐4 in combination with IL‐6 enhanced ALP activity and bone nodule formation by hASCs under normoxia and hypoxia

Subsequently the effect of IL‐4 in combination with IL‐6 on osteogenic differentiation and VEGF expression by hASCs cultured under normoxia and hypoxia was analyzed. The combination of IL‐4 with IL‐6 decreased RUNX2 expression by 1.6–1.9‐fold under hypoxia at Day 2 and 7, as well as under normoxia by 5.6‐fold at Day 7 (Figure 3c,d). Similar to RUNX2, IL‐4 in combination with IL‐6 decreased COL1 gene expression in hASCs by 1.9‐fold under normoxia and 2.2‐fold under hypoxia at Day 7 (Figure 3e). IL‐4 with IL‐6 decreased osteocalcin expression by 1.7‐fold at Day 2 only, while strongly enhancing osteocalcin expression by 5.8‐fold at Day 7 under normoxia. Osteocalcin expression was also enhanced by 1.2‐fold at Day 2 and by 3.8‐fold at Day 7 under hypoxia (Figure 3f,g). Furthermore, ALP activity in hASCs was enhanced by the combination of cytokines by 2.4‐fold under normoxia, and by 12.7‐fold under hypoxia at Day 7 (Figure 3h), while IL‐4 alone reduced ALP activity under normoxia and hypoxia at Day 7 (Figure 1h). The stimulatory effect of IL‐4 in combination of IL‐6 on osteogenic differentiation was also observed by the enhanced mineralization of hASCs under normoxia (6.4‐fold), and by a lesser extend under hypoxia at Day 14 (Figure 3i,j), while IL‐4 alone decreased mineralization of hASCs under hypoxia at Day 14 (Figure 1i,j).

### 3.5 IL‐4 alone, but not in the presence of IL‐6, decreased angiogenic stimulation potential of hASCs under normoxia and hypoxia

**Figure 4:**
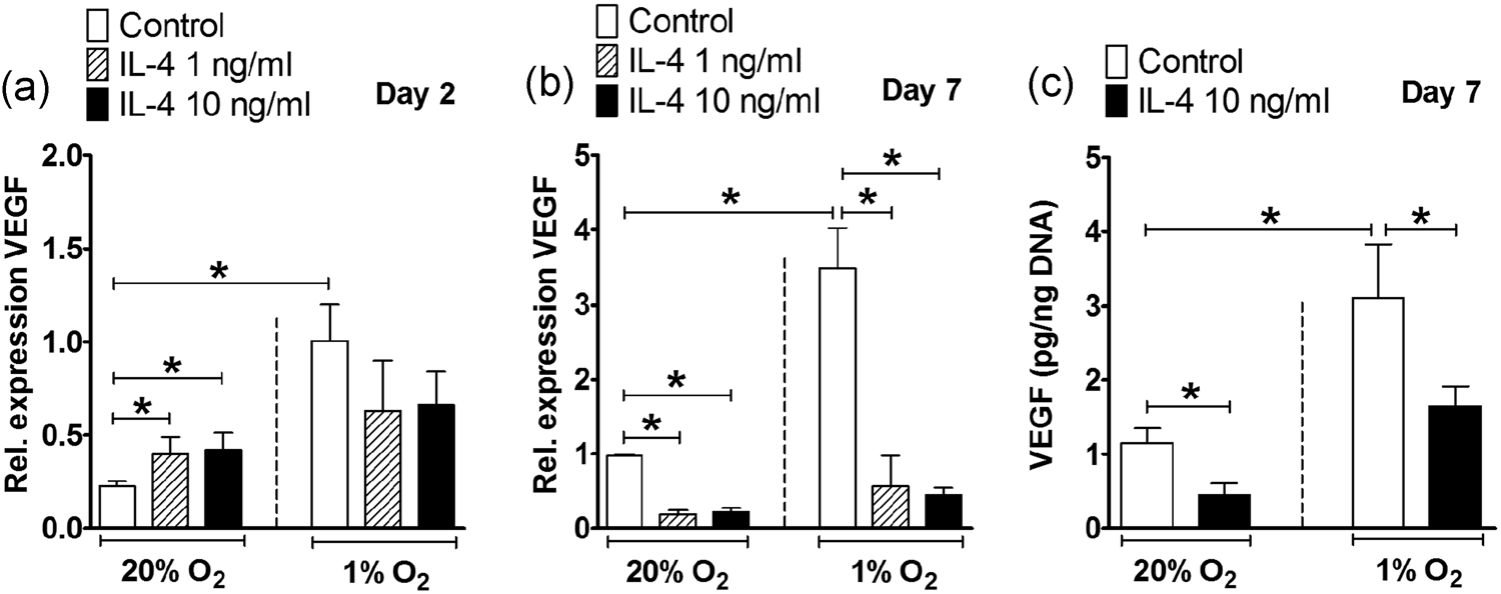
Effect of IL‐4 on VEGF gene and protein expression in hASCs under normoxia or hypoxia. (a,b) VEGF gene expression. Hypoxia enhanced VEGF expression in hASCs at Day 2 and 7. (a) Under normoxia, IL‐4 (1 and 10 ng/ml) increased VEGF expression at Day 2. (b) IL‐4 (1 and 10 ng/ml) decreased VEGF expression under normoxia and hypoxia at Day 7. (c) VEGF protein concentration. Hypoxia enhanced VEGF protein concentration. IL‐4 (10 ng/ml) inhibited VEGF protein independent of the oxygen concentration at Day 7. Results are mean ± *SD* from *n* = 3 (duplicate wells of three independent experiments). *Significant effect of IL‐4 under normoxia or hypoxia, *p* < 0.05. hASC: human adipose stem cell; IL: interleukin; VEGF: vascular endothelial growth factor

IL‐4 (1 and 10 ng/ml) increased VEGF gene expression 1.7–1.9**‐**fold) under normoxia at Day 2 (Figure 4a), but strongly decreased VEGF expression at Day 7 5.1–6.1**‐**fold, 1 ng/ml; 4.4–7.9‐fold, 10 ng/ml) under normoxic and hypoxic culture conditions (Figure 4b). This inhibitory effect of IL‐4 on VEGF was also observed by decreased VEGF protein expression independent of the oxygen concentration at Day 7 (Figure 4C).

**Figure 5:**
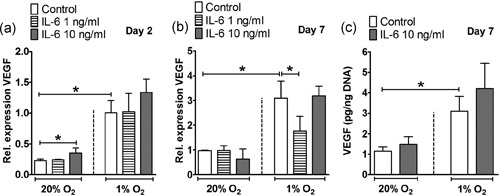
Effect of IL‐6 on VEGF gene and protein expression in hASCs under normoxia or hypoxia. (a,b) Hypoxia enhanced VEGF expression in hASCs at Day 2 and 7. (a) IL‐6 at 10 ng/ml enhanced VEGF expression under normoxia at Day 2. (b) IL‐6 at 1 ng/ml decreased VEGF expression under hypoxia at Day 7. (c) Hypoxia but not IL‐6, enhanced VEGF protein concentration. Results are mean ± *SD* from *n* = 3 (duplicate wells of three independent experiments). *Significant effect of IL‐6 under normoxia or hypoxia, *p* < 0.05. hASC: human adipose stem cell; IL: interleukin; VEGF: vascular endothelial growth factor [Color figure can be viewed at wileyonlinelibrary.com]

IL‐6 (10 ng/ml) increased VEGF gene expression by 1.5‐fold under normoxia at Day 2, while it decreased VEGF expression by 1.7‐fold (1 ng/ml) under hypoxia at Day 7. IL‐6 did not affect VEGF protein expression at Day 7 (Figure 5a–c).

**Figure 6:**
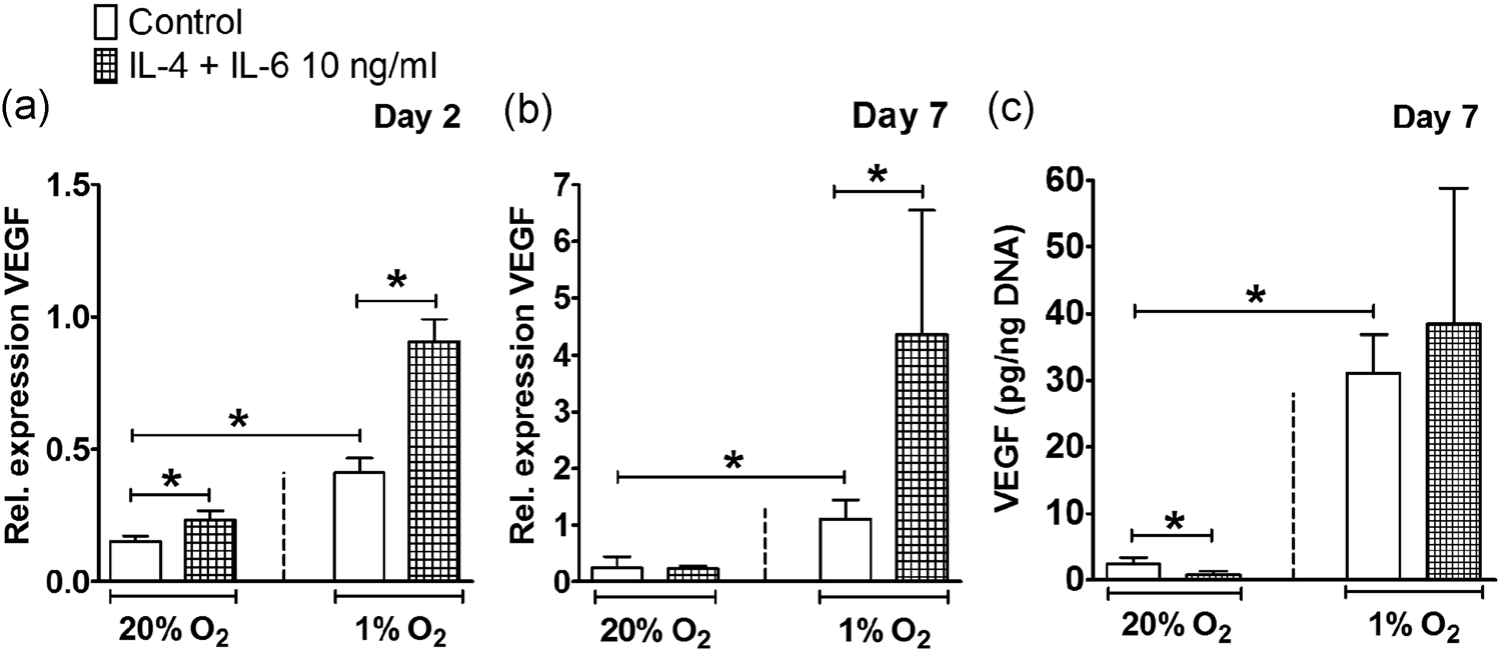
Effect of IL‐4 in combination with IL‐6 on VEGF under normoxia or hypoxia. (a,b) Hypoxia enhanced VEGF expression in hASCs at Day 2 and 7. (a) IL‐4 in combination with IL‐6 increased VEGF expression under normoxia and hypoxia at Day 2. (b) Under hypoxia, IL‐4 in combination with IL‐6 increased VEGF expression at Day 7. (c) At Day 7, hypoxia increased VEGF protein concentration and IL‐4 in combination with IL‐6 decreased VEGF protein concentration under normoxia. Results are mean ± *SD* from *n* = 3 (triplicate wells of three independent experiments). *Significant effect of IL‐4 in combination with IL‐6 under normoxia or hypoxia, *p* < 0.05. hASC: human adipose stem cell; IL: interleukin; VEGF: vascular endothelial growth factor

IL‐4 in combination with IL‐6 enhanced VEGF expression (1.5‐fold) under normoxia at Day 2, and under hypoxia (2.1‐fold) at Day 2, and Day 7 (3.9‐fold; Figure 6A,B). Furthermore, IL‐4 in combination with IL‐6 decreased VEGF protein expression by 3.0‐fold under normoxia, but not under hypoxia at Day 7 (Figure 6c), while IL‐4 alone strongly decreased VEGF under normoxia and hypoxia at Day 7 (Figure 4b).

### 3.6 IL‐4 in combination with IL‐6 enhanced phosphorylation of P70S6K in hASCs under hypoxia

**Figure 7:**
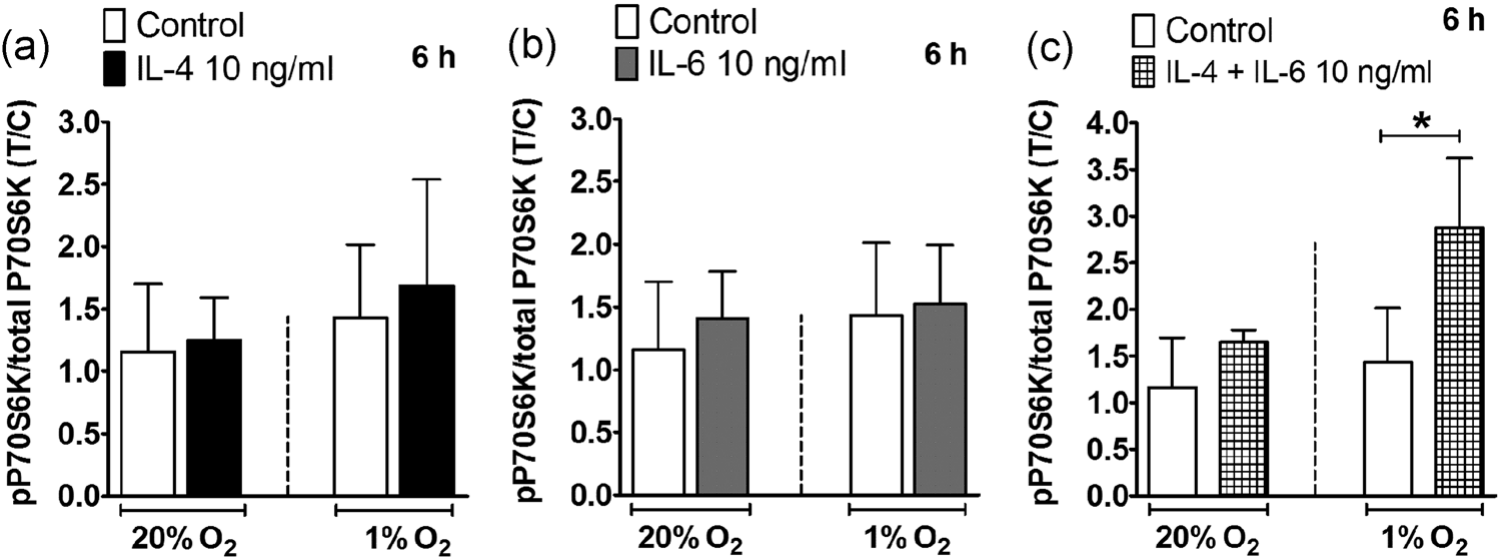
Effect of IL‐4 on the mTOR pathway under normoxia or hypoxia. (a) Phosphorylation of P70S6K in hASCs stimulated with IL‐4 was not affected under normoxia or hypoxia after 6 hr. (b) Phosphorylation of P70S6K in hASCs stimulated with IL‐6 was not affected under normoxia or hypoxia after 6 hr. (c) hASCs stimulated with IL‐4 in combination with IL‐6 increased phosphorylation of P70S6K in hASCs under hypoxia after 6 hr. Results are mean ± *SD* from *n* = 3 (duplicate wells of three independent experiments). *Significant effect of IL‐4, IL‐6, and IL‐4 in combination with IL‐6 under normoxia or hypoxia, *p* < 0.05. hASC: human adipose stem cell; IL: interleukin; mTOR: mammalian target of rapamycin

IL‐4 (Figure 7a), IL‐6 (Figure 7b) did not affect phosphorylation of P70S6K, a downstream effector protein of mTORC1, under any oxygen tension tested. Six hours after cytokine treatment, IL‐4 combined with IL‐6 significantly enhanced phosphorylation of P70S6K (2.8‐fold) in hASCs under hypoxia, but not under normoxia (Figure 7c).

Taken together, IL‐4 did not decrease osteogenic differentiation of hASC, or VEGF expression when applied in combination with IL‐6 under normoxia and hypoxia. Interestingly, IL‐4 in combination with IL‐6 enhanced ALP activity and VEGF expression under normoxia and hypoxia, and stimulated phosphorylation of P70S6K under hypoxia while neither cytokine alone elicited such an effect.

## 4 DISCUSSION

The physiological environment of bone fracture during the initial repair phase is characterized by hypoxia, where different proinflammatory and anti‐inflammatory cytokines are expressed. The hypoxia and cytokine‐activated pathways, such as mTORC1, may have additive or synergistic effects on the osteogenic and angiogenic potential of MSCs. A better understanding of the mechanism of bone healing may lead to the development of new strategies to aid bone repair, which may have implications for the treatment of large bone defects. In the current study we stimulated hASCs with IL‐4, IL‐6, or their combination for 3 days under hypoxia (O_2_, 1%), which simulates the inflammatory environment during early stages of fracture healing in vivo, and also under standard normoxic culture conditions (O_2_, 20%). We investigated the effects of IL‐4 and IL‐6 separately on osteogenic differentiation and angiogenic potential of MSCs and the activation of mTORC1 signaling pathway. Thereafter we investigated the effect of the combination of these two cytokines, with the hypothesis that IL‐6 would counterbalance any inhibitory effects of IL‐4 on hASCs.

Progenitor cells are recruited to the fracture site during the initial stages of fracture healing, where they proliferate in a hypoxic environment (Marsell & Einhorn, 2011). We found that hypoxia inhibited hASC proliferation, observed by decreased DNA content in hASCs cultured without cytokines by 1.3–2.4‐fold at Day 2 and 7 (Figures 1a,b and 3a,b). This is in agreement with findings by others showing reduced hASCs proliferation under hypoxia (O_2_, 1%; Leegwater, Bakker, Hogervorst, Nolte, & Klein‐Nulend, 2017; van Esterik, Jma, P, & Klein‐Nulend, 2017). In addition, previous studies by our group have demonstrated an inhibitory effect of hypoxia on the osteogenic differentiation of MSCs, that is, hASCs cultured under hypoxia for 12 and 21 days showed decreased osteogenic differentiation potential compared with normoxia with a significant reduction in ALP activity (van Esterik et al., 2017; Weijers et al., 2011). In addition, decreased ALP activity in combination with increased gene expression of late osteogenic markers has been reported in human BMMSCs under hypoxia (Lee et al., 2015). These findings suggest that an hypoxic environment might alter the timing of sequential gene expression of early osteogenic differentiation markers in relation to late osteogenic markers during the osteogenic differentiation process (Lee et al., 2015). This is in partial accordance with our results showing that hypoxia decreased ALP activity in hASCs cultured without cytokines by approximately 3.0‐fold at Day 7 compared with normoxia (Figures 1h and 3h), but not mineral deposition by hASCs.

Expression of the cytokines IL‐4 and IL‐6 is significantly elevated in the fracture callus (Einhorn et al., 1995). A recombination activating gene 1 knockout (RAG1^−/−^) mouse lacking the adaptive immune system, thereby having reduced IL‐4 levels, does not show impaired fracture healing (Toben et al., 2011), suggesting that the presence of IL‐4 is not essential for modulating MSC behavior. In contrast, another study has reported that BMMSCs from FBN1‐deficient (Fbn1^+/−^) mice exhibit decreased osteogenic differentiation, and that this lineage alteration is regulated by IL4/IL4Rα‐mediated activation of mTOR signaling to downregulate RUNX2 (Chen et al., 2015). This is in partial accordance with our results showing that IL‐4 strongly reduced RUNX2 and COL1 gene expression as well as ALP activity and mineralization under normoxia and hypoxia. Thus, IL‐4 may inhibit osteogenic differentiation of MSCs and delay fracture healing in vivo. However, we did not find an effect of 6 hr stimulation with IL‐4 on mTOR activation in ASCs, possibly due to the time point chosen or the IL‐4 dose applied. Knowledge about the physiological concentration of cytokines is limited. In general, it is known that the physiological concentration of cytokines is in the order of pg/ml (Hoff et al., 2016). However, it is difficult to measure the exact cytokine concentration after bone fracture, due to the complexity of the fracture hematoma environment, and the level of cytokine variation at different periods of time. In addition, cytokine concentration can vary among individuals (Walters, Pountos, & Giannoudis, 2018). The cytokine concentrations presented in our study can be used as a reference point for relatively high and low cytokine concentration levels. However, our study shows that the effect exerted by IL‐4 or IL‐6 at 1 or 10 ng/ml on osteogenic differentiation and VEGF production in hASCs is not cytokine‐dose dependent. Future research analyzing cytokine expression and concentration during fracture healing in large bones as well as in the skull is needed.

In contrast to the effects of IL‐4, IL‐6^−/−^ mice show delayed callus mineralization and remodeling compared with wild‐type mice, 2 weeks postfracture, indicating that IL‐6 signaling plays an important role in the early stages of fracture healing (Yang et al., 2007). Whether this is caused by a direct regulatory effect of IL‐6 on MSCs, or any other cell type, or whether this can be explained by IL‐6‐modulated effects of other signaling molecules remains to be elucidated. We showed that IL‐6 enhanced mineralization of hASCs under normoxia, which is in agreement with our previous study where IL‐6 also induced mineralization of hASCs under normoxic culture conditions (Bastidas‐Coral et al., 2016). Therefore, IL‐6 may play an important role in the osteogenic differentiation of MSCs.

During the inflammatory phase of fracture healing, cytokines are expressed simultaneously, which may cause synergistic or antagonistic effects in a hypoxic environment. Therefore, we evaluated the combined effect of IL‐4 and IL‐6 on proliferation, osteogenic differentiation, and angiogenic potential of hASCs. We found that IL‐4 with IL‐6 inhibited expression of the early osteogenic markers RUNX2 and COL1, which is similar to the effect of IL‐4 alone. In addition, IL‐4 combined with IL‐6 enhanced mineralization of hASCs under normoxia, which is similar to the effect of IL‐6 alone. Remarkably, ALP activity and mineralization in hASCs were increased only by the combination of IL‐4 with IL‐6 under normoxia and hypoxia, and the inhibitory effect of IL‐4 was counteracted. A study mimicking the endogenous microenvironment of muscle stem cells (MuSCs), showed that only a combination of four proinflammatory cytokines (IL‐1α, IL‐13, TNF‐α, and interferon‐γ) was able to stimulate MuSC proliferation in vivo upon muscle injury and to promote serial expansion of MuSCs in vitro (Fu et al., 2015). These findings suggest that a crosstalk between cytokine signaling and their downstream signaling pathways may occur upon tissue injury in vivo under hypoxia.

During fracture healing, hypoxia re‐establishes oxygen supply by promoting angiogenesis via VEGF (Leegwater et al., 2017; Schipani, Maes, Carmeliet, & Semenza, 2009), which is a key component of bone repair (Farre‐Guasch et al., 2018; Wang et al., 2011). Our findings also showed that hypoxia strongly enhanced VEGF expression in hASCs by ~4.0‐fold at Day 2 and 7 (Figures 4a,b–6a,b). The stimulating effect of hypoxia on VEGF gene expression was confirmed by an approximately eightfold enhanced VEGF protein concentration at Day 7 (Figures 4c and 6c). Under hypoxia, IL‐4 is proangiogenic in the lung via the VEGF pathway, independent of the STAT6 pathway (Yamaji‐Kegan, Su, Angelini, & Johns, 2009). IL‐6 also increases VEGF expression in human umbilical vein endothelial cells in a model studying angiogenesis in rheumatoid arthritis (Kayakabe et al., 2012). Whether IL‐4 and/or IL‐6 have angiogenic stimulation potential on MSCs under normoxia or hypoxia is unknown. In this study, IL‐4 decreased VEGF expression in hASCs under normoxia and hypoxia, while addition of IL‐6 enhanced VEGF expression, showing that IL‐6 counteracted the inhibitory effect of IL‐4 on VEGF expression. Our results indicate that only the combination of IL‐4 with IL‐6, but not IL‐4 alone favored angiogenesis. Therefore, the presence of IL‐6 may contribute to bone fracture repair in vivo.

Signaling pathways activated by IL‐4, IL‐6, and hypoxia such as mTORC1 signaling pathway may interact in MSCs thereby potentially affecting their osteogenic potential. We showed that only the combination of IL‐4 with IL‐6, but not IL‐4 or IL‐6 alone, activated the mTORC1 pathway in hASCs under hypoxia, and that the inhibitory effects of IL‐4, or enhancing effects of IL‐6 under normoxia or hypoxia, were not accompanied by mTORC1 activation. Elevated IL‐4 receptor expression activates mTOR‐P70S6K signaling in BMMSCs in FBN1‐deficient (Fbn1^+/−^) mice, which inhibits RUNX2 expression and suppresses bone regeneration in vitro and in vivo, implicating that IL4Rα/mTOR is the major signaling pathway contributing to the osteopenic phenotype in Fbn1^+/−^ mice (Chen et al., 2015). In addition, mTOR activation may be required for osteoblast proliferation through IL‐6 (Kozawa, Matsuno, & Uematsu, 2001; Takai et al., 2007; Takai et al., 2008). Further studies are needed to unravel the mechanisms responsible for the effects induced by IL‐4 and IL‐6 and their interaction with mTORC1.

**Figure 8:**
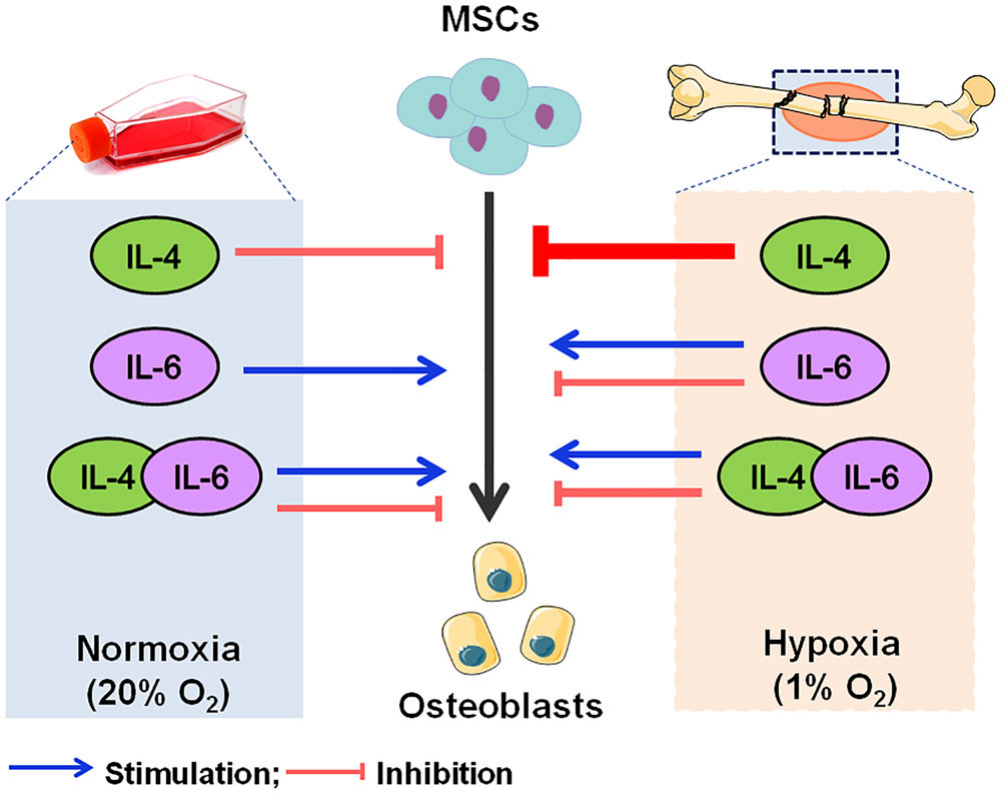
Schematic representation of how IL‐4, IL‐6 and the combination of IL‐4 with IL‐6 might influence osteogenic differentiation of MSCs under normoxia or hypoxia, and how they might contribute to bone repair under hypoxia. IL: interleukin; MSC: mesenchymal stem cell [Color figure can be viewed at wileyonlinelibrary.com]

In conclusion, IL‐4 reduced osteogenic differentiation of hASCs, indicating that this cytokine might inhibit bone healing and regeneration. However, the effects of IL‐4 were mitigated in the presence of IL‐6 (Figure 8). This shows that for a better understanding of bone healing, for example for tissue engineering purposes, it is important to move towards more complex in vitro systems, taking into account factors such as oxygen tension and combinations of cytokines.

## CONFLICT OF INTERESTS

The authors declare that there are no conflict of interests.

## AUTHOR CONTRIBUTIONS

A. P. B.‐C. has made substantial contributions to conception and design, acquisition of data, and analysis and interpretation of data, and been involved in drafting the manuscript and revising it critically for important intellectual content, and given final approval of the version to be published. J. M. A. H. has made substantial contributions to acquisition of data, been involved in drafting the manuscript, and given final approval of the version to be published. T. F. has made substantial contributions to conception and design, and analysis and interpretation of data, been involved in revising the manuscript critically for important intellectual content, and given final approval of the version to be published. C. J. K. has made substantial contributions to conception and design of data, been involved in revising the manuscript critically for important intellectual content, and given final approval of the version to be published. P. K. has made substantial contributions to acquisition of data, been involved in revising the manuscript critically for important intellectual content, and given final approval of the version to be published. J. K.‐N. has made substantial contributions to conception and design and interpretation of data, been involved in drafting the manuscript and revising it critically for important intellectual content, and given final approval of the version to be published. A. D. B. has made substantial contributions to conception and design, and analysis and interpretation of data, and been involved in drafting the manuscript and revising it critically for important intellectual content, and given final approval of the version to be published. A. P. B.‐C., J. M. A. H., T. F., C. J. K., P. K., J. K.‐N., and A. D. B. have participated sufficiently in the work to take public responsibility for appropriate portions of the content, and agreed to be accountable for all aspects of the work in ensuring that questions related to the accuracy or integrity of any part of the work are appropriately investigated and resolved.
